# Molecular Mechanism of Zinc-Dependent Oligomerization of Alzheimer’s Amyloid-β with Taiwan (D7H) Mutation

**DOI:** 10.3390/ijms241411241

**Published:** 2023-07-08

**Authors:** Olga I. Kechko, Alexei A. Adzhubei, Anna P. Tolstova, Maria I. Indeykina, Igor A. Popov, Sergey S. Zhokhov, Nikolay V. Gnuchev, Vladimir A. Mitkevich, Alexander A. Makarov, Sergey A. Kozin

**Affiliations:** 1 Engelhardt Institute of Molecular Biology, Russian Academy of Sciences, 119991 Moscow, Russia; olga.kechko@gmail.com (O.I.K.); alexei.adzhubei@gmail.com (A.A.A.); tolstova.anna.pavlovna@gmail.com (A.P.T.); mariind@yandex.ru (M.I.I.); nikolay.gnuchev@yahoo.com (N.V.G.); aamakarov@eimb.ru (A.A.M.); 2 Emanuel Institute of Biochemical Physics, Russian Academy of Sciences, 119334 Moscow, Russia; 3 Moscow Institute of Physics and Technology, 141701 Dolgoprudny, Russia; hexapole@gmail.com; 4 Department of Chemistry, M.V. Lomonosov Moscow State University, 119991 Moscow, Russia; szhokhov@gmail.com

**Keywords:** Alzheimer’s disease, amyloid-beta, familial Taiwan mutation D7H, zinc, metal binding domain, oligomerization, aggregation seeding, drug target, amyloid plaque formation

## Abstract

Amyloid-β (Aβ) is a peptide formed by 39–43 amino acids, heterogenous by the length of its C-terminus. Aβ constitutes a subnanomolar monomeric component of human biological fluids; however, in sporadic variants of Alzheimer’s disease (AD), it forms soluble neurotoxic oligomers and accumulates as insoluble extracellular polymeric aggregates (amyloid plaques) in the brain tissues. The plaque formation is controlled by zinc ions; therefore, abnormal interactions between the ions and Aβ seem to take part in the triggering of sporadic AD. The amyloid plaques contain various Aβ isoforms, among which the most common is Aβ with an isoaspartate in position 7 (isoD7). The spontaneous conversion of D7 to isoD7 is associated with Aβ aging. Aβ molecules with isoD7 (isoD7-Aβ) easily undergo zinc-dependent oligomerization, and upon administration to transgenic animals (mice, nematodes) used for AD modeling, act as zinc-dependent seeds of the pathological aggregation of Aβ. The formation of zinc-bound homo- and hetero-oligomers with the participation of isoD7-Aβ is based on the rigidly structured segment 11-EVHH-14, located in the Aβ metal binding domain (Aβ_16_). Some hereditary variants of AD are associated with familial mutations within the domain. Among these, the most susceptible to zinc-dependent oligomerization is Aβ with Taiwan (D7H) mutation (D7H-Aβ). In this study, the D7H-Aβ metal binding domain (D7H-Aβ_16_) has been used as a model to establish the molecular mechanism of zinc-induced D7H-Aβ oligomerization through turbidimetry, dynamic light scattering, isothermal titration calorimetry, mass spectrometry, and computer modelling. Additionally, the modeling data showed that a molecule of D7H-Aβ, as well as isoD7-Aβ in combination with two Aβ molecules, renders a stable zinc-induced heterotrimer. The trimers are held together by intermolecular interfaces via zinc ions, with the primary interfaces formed by 11-EVHH-14 sites of the interacting trimer subunits. In summary, the obtained results confirm the role of the 11-EVHH-14 region as a structure and function determinant for the zinc-dependent oligomerization of all known Aβ species (including various chemically modified isoforms and AD-associated mutants) and point at this region as a potent target for drugs aimed to stop amyloid plaque formation in both sporadic and hereditary variants of AD.

## 1. Introduction

The oligomerization and aggregation of human amyloid-beta (Aβ) play a crucial role in the development of Alzheimer’s disease (AD) [1]. The formation and spread of extracellular Aβ aggregates (amyloid plaques) in brain tissues is one of the main pathomorphological features of AD and, possibly, the primary pathogenic process of AD [2]. Therefore, inhibition of the formation of Aβ oligomers and aggregates is viewed as one of the most promising strategies in the development of disease-modifying therapeutic approaches in AD [3]. A fragment 1–16 of Aβ amino acid sequence (Aβ_16_) is routinely used as the most suitable drug target for monoclonal antibodies in the implementation of this approach [4]. However, monoclonal antibodies have many side effects [5]; therefore, the search for shorter functional Aβ fragments that can be used as drug targets for small molecular weight agents that specifically bind to Aβ through the targeted site and prevent the formation and spread of Aβ oligomers and/or aggregates remains highly relevant [6].

A number of observations indicate the possible involvement of Aβ interactions with zinc ions in the pathogenesis of AD [7]: (1) amyloid plaques contain abnormally high amounts of zinc ions [8]; (2) zinc binds to Aβ and causes its rapid aggregation [9], presumably due to the modulation of Aβ’s conformational transformation and the population shift of the equilibrium of polymorphic Aβ states [10]; (3) in post-mortem brain tissue samples of patients diagnosed with AD, areas of elevated zinc concentration coincide with the sites of amyloid plaque formation [11]; (4) the areas of the brain most affected by AD pathology contain a dense innervation of zinc-containing axons, while areas of the brain less affected by this pathology contain insignificant amounts of zinc-containing endings [12].

Amyloid plaques are formed in brain tissues through a seeding aggregation mechanism [13], and it is impossible without the participation of zinc ions (reviewed in [14]). Aβ_16_ is a metal-binding domain that forms an equimolar complex upon interaction with a zinc ion [15,16]. The spatial structure of such a complex produces an increase in its integral hydrophobicity and, as a result, enhances the tendency of zinc-bound Aβ species to oligomerization and aggregation compared to zinc free Aβ species in in vitro experiments [17]. However, oligomers and aggregates of both free and zinc-bound Aβ species do not affect the development of AD [18].

Interspecies differences, genetic mutations, and/or chemical modifications of amino acid residues in Aβ_16_ significantly alter the molecular mechanisms of the interaction of the corresponding Aβ isoforms with zinc ions (reviewed in [14]). For example, Aβ_16_ maintains its monomeric state upon zinc binding [16,19], whereas Aβ_16_ molecules with an isomerized aspartate in position 7 (isoD7-Aβ_16_), which is the most frequently occurring Aβ modification in amyloid plaques [20], form zinc-induced oligomers, where zinc ions are located at the intermolecular interfaces of the interacting subunits [21]. It is important to note that unlike Aβ, isoD7-Aβ and isoD7- Aβ_16_ act as exogenous triggers of amyloid plaques formation in mouse models of AD [22,23]. Using transgenic nematodes *C. elegance* as an AD model, it was shown that zinc-bound isoD7-Aβ molecules are a necessary and sufficient agent that causes the aggregation of endogenous Aβ molecules [24]. It was suggested that zinc-bound isoD7-Aβ oligomers can act as “amyloid matrices”, upon contact with which Aβ molecules lose their monomeric conformation and undergo further aggregation [6].

Along with post-translational modifications of Aβ, hereditary mutations were found in some patients, some of which lie in the region of the metal binding domain 1–16 of Aβ molecules [25]. Such mutations affect the toxicity and rate of Aβ oligomerization in the presence of metals, causing the development of early onset AD. Among them, Aβ with Taiwan (D7H) mutation (D7H-Aβ) has the highest aggregation ability [26]. D7H-Aβ is associated with early-onset familial AD [27,28]. This mutation shifts the processing of amyloid precursor protein from the non-amyloidogenic to the amyloidogenic pathway, increases the Aβ_42_/Aβ_40_ ratio in the body, increases the Aβ affinity for zinc ions, and contributes to the stabilization of the oligomeric state of the peptide, which causes its high neurotoxicity [27,28]. The N-terminal part of D7H-Aβ features a unique binuclear zinc ion binding site formed by the amino acid residues D1, E3, H6, and H7. This site may be responsible for the superior ability of D7H-Aβ to form zinc-induced oligomers/aggregates compared with all other known Aβ isoforms [26].

Using synthetic analogs of the metal binding domain of various amyloid-beta isoforms, it was previously shown that stable zinc-dependent dimers, in which one zinc ion is located at the 11-EVHH-14 intermolecular interface of the interacting peptide subunits, are formed by rat amyloid-beta (ratAβ) (containing three amino acid substitutions: R5G, Y10F, and H13R) [29]; by the AD-associated Aβ isoform with the English (H6R) mutation (H6R-Aβ) [30]; by Aβ with a phosphorylated Ser8 residue (pS8-Aβ) [31]; and by isoD7-Aβ [21]. At the same time, Aβ_16_ does not form such dimers [15,16]. In contrast to all of the other Aβ isoforms mentioned above, isoD7-Aβ_16_ in the presence of zinc ions undergoes zinc-dependent oligomerization, where, in addition to the primary dimerization interface 11-EVHH-14, a second zinc-mediated oligomerization interface is formed by a pair of H6 and H13 residues [21]. It was also shown that, unlike Aβ_16_, isoD7-Aβ_16_, upon intracerebral administration to model animals, sharply accelerates the amyloid plaque formation, suggesting that zinc-induced isoD7-Aβ_16_ oligomers represent minimal seeds for the aggregation of Aβ molecules [23].

In this study, we applied turbidimetry, dynamic light scattering (DLS), isothermal titration calorimetry (ITC), mass spectrometry, and computer simulation to establish the molecular mechanism of zinc-induced D7H-Aβ oligomerization and to reveal the role of the 11-EVHH-14 region as a structure and function determinant of this process. The findings confirm this region as a potential drug target for the development of disease-modifying therapy in the pathogenesis of AD, based on the selective suppression of the formation of pathogenic zinc-dependent seeds of Aβ aggregation [24,32]. Also, proceeding from the obtained results, it was shown that both isoD7-Aβ and D7H-Aβ form zinc-bound heterotrimers with two Aβ molecules. It is these trimers that probably represent the minimal aggregation seeds in sporadic AD variants accompanied with the appearance of isoD7-Aβ, as well as in the hereditary Taiwan variant of AD. Further, analysis of the similarities and differences in the structural organization of trimers that incorporate D7H-Aβ and isoD7-Aβ suggests that intravenous injections of synthetic D7H-Aβ peptide are likely to cause a much stronger amyloidogenic effect in animal models of AD compared to the effect observed for an isoD7-Aβ peptide.

## 2. Results

### 2.1. Aggregation States of Aβ_16_ and D7H-Aβ_16_ in the Presence of Zinc Ions

**Figure 1:**
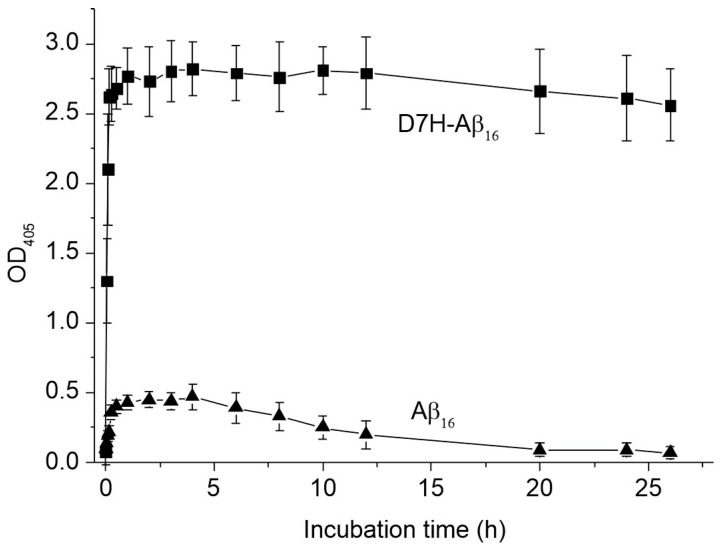
Dependence of turbidity (optical density at 405 nm, OD_405_) of Aβ_16_ (black triangles) and D7H-Aβ_16_ (black squares) solutions (1 mM) on the incubation time after the addition of Zn^2+^ (2.25 mM). Measurements were performed in 50 mM Tris buffer, pH 7.3. The means and standard deviations for three measurements are shown.

Turbidity measurements is one of the classic methods for assessing the aggregate state of amyloid-beta peptides and their fragments under various experimental conditions [33]. Turbidimetry was used for a comparative assessment of the aggregation state of 1 mM aqueous solutions of D7H-Aβ_16_ and Aβ_16_. The measurements were conducted in the presence of equimolar amounts of zinc ions in order to identify the integral differences in the aggregation ability of these peptides and their zinc complexes. In the absence of zinc ions, there was no change in the turbidity of the peptides D7H-Aβ_16_ and Aβ_16_ solutions for at least 2 h. The addition of Zn^2+^ to D7H-Aβ_16_ triggered a rapid peptide aggregation, manifested by a rise in the solution turbidity (Figure 1). The maximal turbidity of the Aβ_16_ solution in the presence of Zn^2+^ was 5–6 times lower than for D7H-Aβ_16_. The turbidity signal of D7H-Aβ_16_ was stable for 26 h after the addition of ZnCl_2_. In contrast, within the same timeframe, the turbidity of the Aβ_16_ solution decreased by 6–8 times. These data indicate that after the addition of zinc ions to Aβ_16_, zinc-bound monomeric complexes Zn/Aβ_16_ are formed, together with a minor fraction of metastable oligomers, which subsequently decompose to the corresponding monomers within a 26 h period. In the absence of zinc ions, for free D7H-Aβ_16_, the turbidity signal practically corresponds to that of Aβ_16_, confirming the monomeric status of D7H-Aβ_16_ under these conditions. The addition of zinc ions to the D7H-Aβ_16_ solution did not lead to the precipitation of the peptide, as evidenced by a stable turbidity value over time.

**Figure 2:**
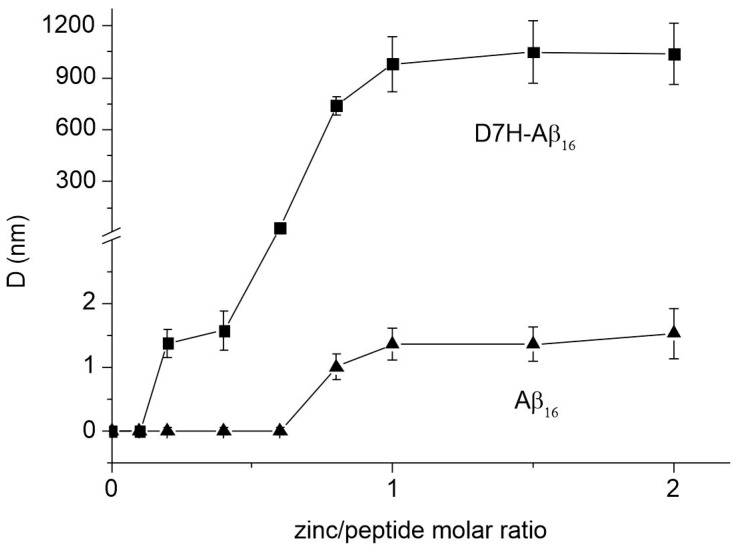
The characteristic diameter (D) of Zn^2+^-induced Aβ soluble aggregates as a function of the Zn^2+^/peptide molar ratio. D values of Aβ_16_ (black triangles) and D7H-Aβ_16_ (black squares) soluble aggregates were measured after 10-min incubation with Zn^2+^ in 50 mM Tris buffer, pH 7.3. Concentration of peptides was 50 µM. The means and standard deviations for three measurements are shown.

DLS was used to characterize the sizes of the zinc-dependent Aβ_16_ and D7H-Aβ_16_ soluble aggregates (Figure 2). In the absence of zinc ions, soluble aggregates in the solutions of Aβ_16_ and D7H-Aβ_16_ peptides were not detected. Soluble aggregates of Aβ_16_ started to be detected at a Zn/peptide ratio of 0.8. The maximum size of the Aβ_16_ soluble aggregates in the presence of a twofold excess of Zn ions was 1.5 nm. The same soluble aggregate size is observed for D7H-Aβ_16_ at a Zn/peptide ratio of 0.2. An increase in this ratio above 0.5 leads to a rapid increase in the size of the zinc-dependent D7H-Aβ_16_ soluble aggregates. At an equimolar Zn/peptide ratio, the characteristic diameter of the soluble aggregates reached a size of about 1000 nm and then did not change. Overall, the results of the DLS and turbidimetry show that the Taiwan mutation (D7H) in Aβ_16_ leads to a dramatic change in the aggregation properties of the peptide, which may be due to a change in its interaction with Zn ions.

### 2.2. Determination of the Thermodynamic Parameters of the Formation of Zinc-Bound Complexes of Mutant Aβ_16_ Isoforms by Isothermal Titration Calorimetry (ITC)

**Figure 3:**
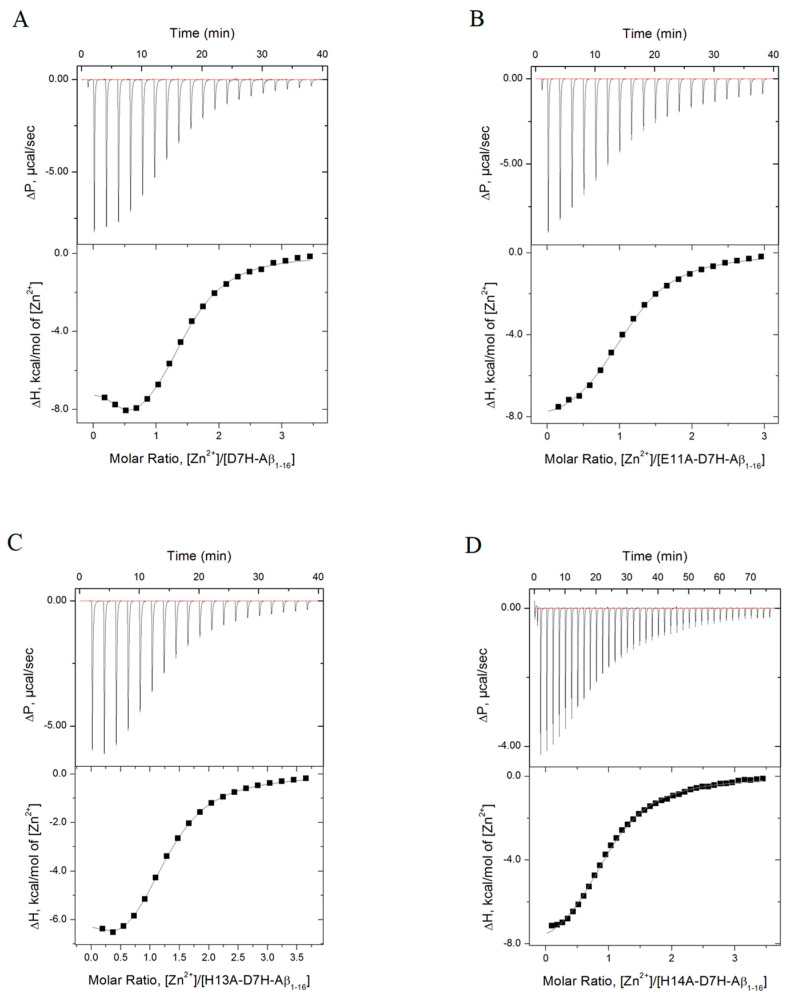
ITC titration curve (upper panel) and the binding isotherm (lower panel) for Zn^2+^ (5 mM) interaction with 0.3 mM D7H-Aβ_16_ (**A**), E11A-D7H-Aβ_16_ (**B**), H13A-D7H-Aβ_16_ (**C**), H14A-D7H-Aβ_16_ (**D**) at 25 °C in 50 mM Tris buffer, pH 7.3.

ITC was used to analyze zinc-dependent interactions of the intact D7H-Aβ_16_ peptide and its mutants, where the amino acid residues that play a role in binding the zinc ion to the D7H-Aβ_16_ peptide, were replaced by alanine residues. The curve of the D7H-Aβ_16_ binding isotherm with the zinc ions showed the presence of more than one Zn^2+^ binding site. Indeed, this isotherm can be well fitted using a model of two interaction sites (Figure 3A, Table 1). The association constants and enthalpy changes are comparable during the interaction of mutant peptides with zinc ions in comparison with the intact D7H-Aβ_16_ (Table 1). The enthalpy change, as a result of forming such zinc–peptide complexes, is negative; therefore, the interaction is enthalpy-driven. The stoichiometry of the low-affinity binding site of D7H-Aβ_16_ with Zn^2+^ is 1. A comparison of the binding parameters on zinc binding by Aβ_16_, D7H-Aβ_16_, and D7H-Aβ_10_ indicates a localization of the low-affinity binding site in the region 1–10 (Aβ_10_) (Table 1). We previously showed that D7H-Aβ_10_ is capable of forming a stable antiparallel dimer that binds two zinc ions (stoichiometry 2:2) [26]. Zinc ions in this dimer are coordinated by the amino acid residues Asp1, Glu3, His6, and His7 of each peptide. The stoichiometry of the high affinity site (N = 0.35) located at the C-terminus of the peptide D7H-Aβ_16_ signifies the formation of higher order oligomers. According to the published data, the Aβ region 11-EVHH-14 is not only involved in the chelation of Zn^2+^, but also serves as an interface for the zinc-induced dimerization of the metal-binding domain of Aβ isoforms [21,31,34,35]. To identify the amino acid residues involved in the coordination of zinc ions through D7H-Aβ_16_, we used D7H-Aβ_16_ mutants carrying substitutions of Glu11, His13, or His14 for Ala (Table 1). Mutations at amino acids 11 and 14 led to the complete shutdown of the high affinity site 11-EVHH-14 (Figure 3B,D, Table 1). Substitution H13A does not substantially affect the interaction of D7H-H13A-Aβ_16_ with Zn^2+^; the finding that excludes the involvement of amino acid residue H13 in the high-affinity interaction interface (Figure 3C, Table 1). Hence, our ITC data show that the binding of zinc ions to D7H-Aβ_16_ leads to the formation of oligomers that lack the binuclear zinc ion binding site observed for D7H-Aβ fragments 1–7 (D7H-Aβ_7_) and 1–10 (D7H-Aβ_10_), but features at least two potential zinc-mediated interfaces: the high affinity Aβ region 11–14 and low affinity Aβ region 1–7. Further, it was found that H13 does not bear upon the activity of the high-affinity interface, which does not exclude the involvement of this residue in the formation of a low-affinity site comprising amino acid residues 1–7. The absence of the binuclear center indicates the predominant role of site 11–14 in the recognition of zinc ions by the peptide D7H-Aβ_16_.

### 2.3. Detection of Zinc Ion Chelators in the D7H-Aβ_16_ Monomer and Dimer by Mass Spectrometry

To acquire additional information on the involvement of amino acid residues from D7H-Aβ_16_ in the formation of zinc complexes, we used the method of tandem mass spectrometry. This approach previously demonstrated its efficiency in solving a similar problem in the case of Aβ_16_ and the metal-binding domains of other amyloid-beta isoforms [19,26,29,31,36,37,38].

**Figure 4:**
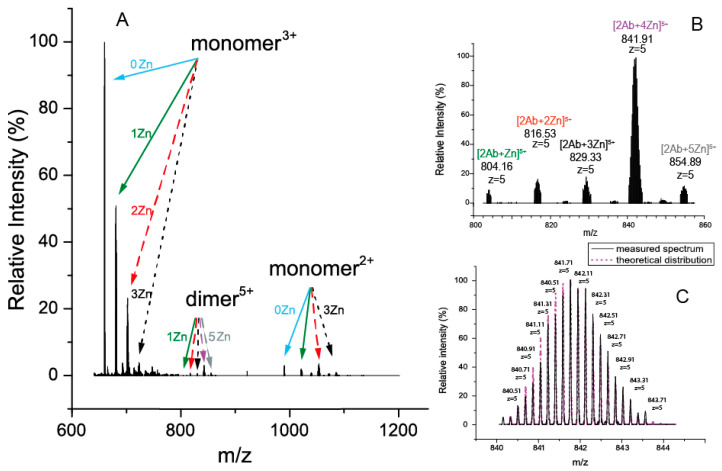
(**A**) Mass-spectrum of complexes D7H-Aβ_16_ with zinc ions (arrows indicate number of Zn^2+^: blue—0, green—1, red—2, black—3, violet—4, grey—5). Monomeric and dimeric complexes with different amounts of zinc adducts are observed. (**B**) Enlargement of a high-resolution FT ICR MS spectrum of the dimer complex cluster region. (**C**) The theoretical and measured isotopic distributions are in good agreement.

**Figure 5:**
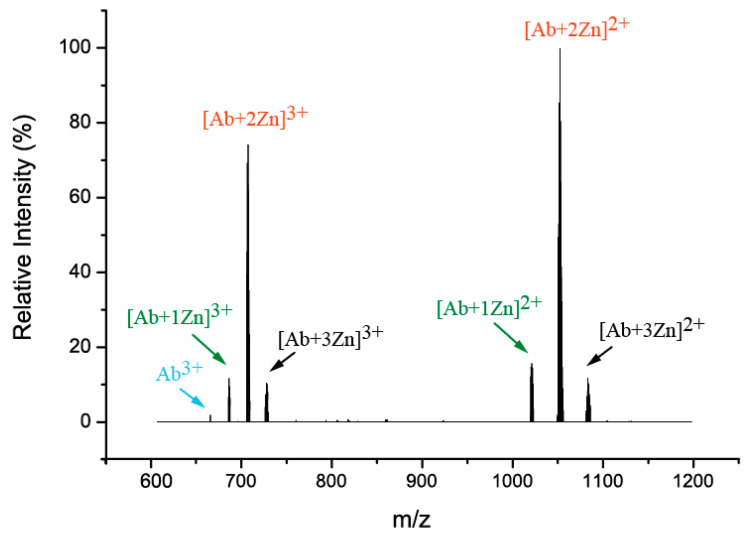
Dissociation of the D7H-Aβ_16_ peptide dimer with 4 zinc ions results in the formation of quasi-molecular monomer ions with different numbers of zinc ions (no peptide fragments are formed).

In the presence of zinc ions, D7H-Aβ_16_ forms stable monomers and dimers with various numbers of metal ions bound in a wide range of charge states (Figure 4). In the process of fragmentation, only quasi-molecular ions of the monomeric peptide with and without zinc ions were formed (Figure 5). A MS3 experiment with these quasi-molecular ions gave spectra that were identical to those usually obtained as a result of the MS/MS fragmentation of a complex represented by the monomer with zinc, also formed under MS conditions. It was concluded that the zinc ion coordinators in a dimer and monomer complexes are similar and, thus, monomers may be used for further study.

**Figure 6:**
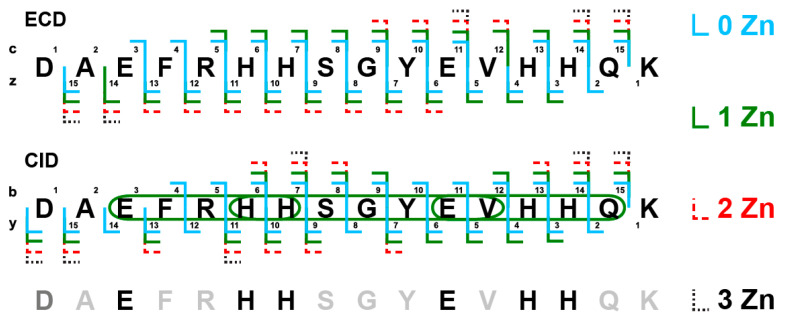
Results of MS/MS analysis of monomer complexes D7H-Aβ_16_ with different amounts of zinc adducts. Ions carrying no, one, two and three zinc ions are shown by solid light blue, solid green, red dashed and black dotted lines, respectively. The smallest internal fragments carrying one zinc ion are circled. The assumed zinc ion chelator candidates are shown in black.

The monomer complexes of the D7H-Aβ_16_ peptide with up to three zinc ions were registered (Figure 6). We anticipate that under ESI conditions, the zinc chelating residues may capture one zinc ion per residue instead of coordinating a single ion jointly, considering the limitations caused by steric and electrostatic hindrances. The monomeric complexes with zinc ions in different charge states were isolated and fragmented using CID and ECD methods. Analysis of the intersections among the fragment ions carrying various amounts of zinc and the capabilities of the amino acid residues in question to chelate zinc ions (of note, for the amino acids such as glycine, alanine and likewise this is chemically not possible) points at the probable chelators. The lack of fragmentation in certain areas also hints at possible zinc localization due to the bridge formation through the ion or the loss of zinc instead of backbone cleavage. To summarize this data, the two glutamate and histidine residues are the most likely candidates to coordinate zinc ions.

Our mass spectrometry results show that in the intermolecular interfaces of D7H-Aβ_16_ zinc-induced oligomers, the preferred amino acid residues involved in the chelation of shared zinc ions are E3, H6, H7, E11, H13, and H14. These data are consistent with the above-described ITC data of this study on the role of E11 and H14 in the formation of a high-affinity interface. Mass spectrometry indicated the four candidates for participation in the low-affinity interface of the zinc-bound D7H- Aβ_16_ oligomers, providing indirect evidence in favor of the existence of not one, but two, low-affinity interfaces. At the same time, the formation of the H6/H13 pair for zinc ion chelation is impossible due to steric hindrance [21]. Therefore, in a zinc-mediated intermolecular interface, the E11/H14 pair should be combined either with the E3/H6 and H7/H13 pairs or the E3/H13 and H6/H7 pairs.

### 2.4. Molecular Modeling of Zinc-Induced Oligomers of D7H-Aβ_16_

Molecular modelling employing Molecular Dynamics (MD) was used to analyze the structure patterns of the zinc-induced oligomers of D7H-Aβ_16_ at the initial stages of aggregation. Trajectory analysis of the 200 ns MD modeling of the D7H-Aβ_16_ dimer bound through the E11/H14:ZN:E11/H14 interface was applied to select the preferable combinations of amino acid residues from the initial set—obtained according to the MS data (Section 2.3)—involved in the chelation of the shared zinc ions inside the zinc-induced oligomers of D7H-Aβ_16_. We conducted both clustering and distance calculations for the pairs of the residues E3, H6, H7, and H13 for each molecule over all frames of the MD trajectory to determine the preferred conformations of the D7H-Aβ_16_ dimer. The analysis showed that residues E3 and H13 over the whole 200 ns MD modeling time never appear within at least a 4.5 Å range from each other. Therefore, the interface based on the E3/H13 and H6/H7 pairs was excluded from the modeling in this study. However, we cannot dismiss the possibility that this interface could emerge in more complex systems with a higher number of molecules than that examined in this study. In the interface based on the pairs E3/H6 and H7/H13, an antiparallel arrangement of the 11-EVHH-14 sites in the peptides brings residues H6/H7 and H13 of the peptides in the dimer close to each other. Pair H7/H13 appeared in 36 frames of 20,000 and pair H6/H13 in 3 frames. Residues E3/H6 and E3/H7 were spatially close to each other in 253 and 2141 frames, respectively. However, pairs E3/H7 and H6/H13 are unlikely to exist concurrently in the same molecule due to steric clashes and were not taken into account here. Overall, the MD modeling showed that the most likely interaction interfaces for the zinc-induced oligomerization of D7H-Aβ_16_ are E11/H14:ZN:E11/H14, E3/H6:ZN:E3/H6, and H7/H13:ZN:H7/H13.

**Figure 7:**
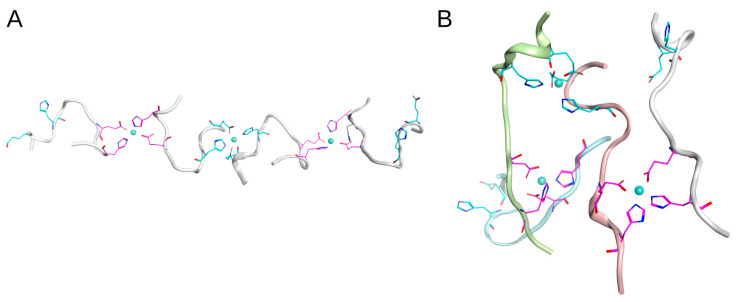
Tetramer constructed with D7H-Aβ_16_ peptides with interlacing E11/H14:ZN:E11/H14 and E3/H6:ZN:E3/H6 interfaces between peptides. (**A**) the initial conformation after energy minimization. (**B**) The final conformation after 50 ns of MD. Residues Glu11 and His14 are colored with magenta. Residue Glu3 and His6 are colored with cyan.

**Figure 8:**
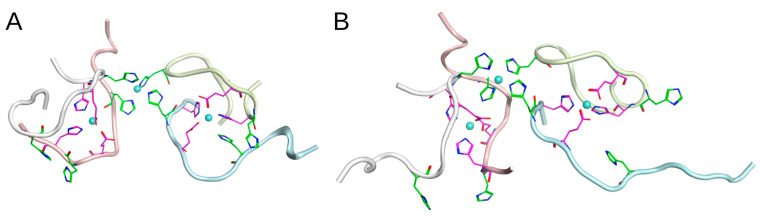
Tetramer constructed with D7H-Aβ_16_ peptides with interlacing E11/H14:ZN:E11/H14 and H7/H13:ZN:H7/H13 interfaces between peptides. (**A**) the initial conformation after energy minimization. (**B**) The final conformation after 50 ns of MD. Residues Glu11 and His14 are colored with magenta. Residue His7 and His13 are colored with green.

The molecular modeling described above has shown that, due to steric constraints, the E3/H13 and H6/H7 pairs cannot participate in the formation of stable zinc-bound D7H-Aβ_16_. Therefore, based on our ITC and MS results (see sections above), we examined the following three different binding interfaces for the D7H-Aβ_16_ peptides involving zinc atoms: E11/H14:ZN:E11/H14, E3/H6:ZN:E3/H6, and H7/H13:ZN:H7/H13. The D7H-Aβ_16_ dimer with a high affinity zinc-mediated interface E11/H14:ZN:E11/H14 was designated as a polymerization seed and the unit for oligomer formation. As a tetramer pattern, two D7H-Aβ_16_ dimer conformations were selected from the MD trajectory with close relative positions of residues E3/H6 or H7/H13 (Figure 7 and Figure 8).

Providing that the E11/H14:ZN:E11/H14 and E3/H6:ZN:E3/H6 interfaces do not overlap and are located at a significant distance from each other, the D7H-Aβ_16_ polymers with alternate interfaces can be formed without hindrance. In Figure 7A, the residues not involved in the peptide interaction are shown, as well as the residues included in the interaction centers. It is clearly seen that the free E3/H6 pairs are located close to each other and can form a new zinc coordinating center with a new peptide. The system constructed with interlacing E11/H14:ZN:E11/H14 and E3/H6:ZN:E3/H6 interfaces, shown in Figure 7A, can be easily extended in the lateral direction through the addition of D7H-Aβ_16_ dimers to the left and to the right in parallel to the already present ones. A 50 ns MD modeling was performed for such a tetramer, showing it as stable. However, the relative location of the D7H-Aβ_16_ peptides has changed and they took a more elongated shape with an antiparallel positioning of the peptides. The residues E3 and H6 remained closely spaced after MD, providing possible seeds for polymerization. We speculate that this variant of D7H-Aβ_16_ polymer can grow, forming stacks of extended D7H-Aβ_16_ peptides with antiparallel packing and the E11/H14:ZN:E11/H14 and E3/H6:ZN:E3/H6 interfaces.

**Figure 9:**
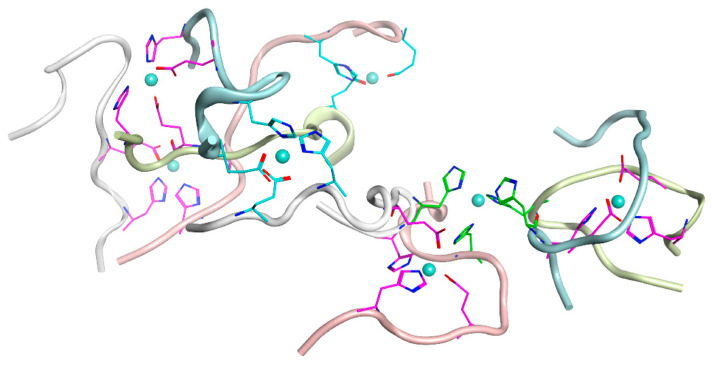
Octamer constructed with D7H-Aβ_16_ peptides with interlacing E3/H6:ZN:E3/H6, E11/H14:ZN:E11/H14 and H7/H13:ZN:H7/H13 interfaces between peptides after 50 ns of MD. Residues Glu11 and His14 are colored with magenta. Residue His7 and His13 are colored with green. Residue Glu3 and His6 are colored with cyan.

**Figure 10:**
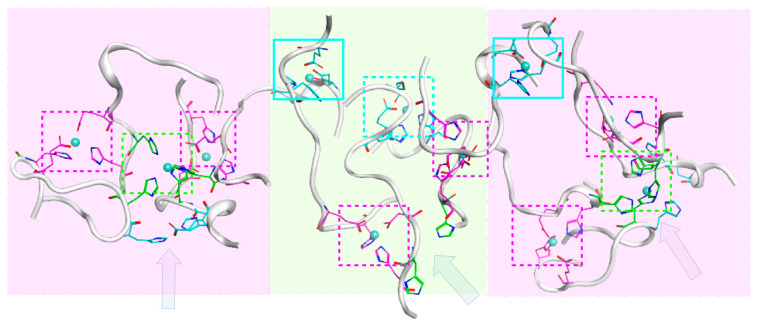
Dodecamer constructed with D7H-Aβ_16_ peptides with interlacing E3/H6:ZN:E3/H6, E11/H14:ZN:E11/H14 and H7/H13:ZN:H7/H13 interfaces between peptides after 50 ns of MD. Residues Glu11 and His14 are colored with magenta. Residue His7 and His13 are colored with green. Residue Glu3 and His6 are colored with cyan. The system was built of three tetramers highlighted with pink for tetramers with the H7/H13:ZN:H7/H13 interface and yellow for tetramer with the E3/H6:ZN:E3/H6 interface. Interaction interfaces are highlighted with dotted squares for the internal interactions inside each tetramer and with solid line squares for interactions between tetramers. The potential seeds for polymerization are shown with arrows.

In the case of the H7/H13:ZN:H7/H13 interface between two D7H-Aβ_16_ dimers, the oligomers formed via such an interface will be subject to additional restrictions on the conformation of each D7H-Aβ_16_ molecule due to the substantial distance between residues H7 and H13 of the peptides and the overlap of the two Zn coordination centers, H7/H13 and E11/H14. In the presence of the Zn ion in the coordinating center H11/H14, residues H7 and H13 of the same peptide are unlikely to be positioned close enough to each other to bind another Zn atom. Even if realized, such a system would be unstable due to the structural tension caused by the interfaces’ concurrence. Although residues H7 and H13 within the peptide are located far from each other, residues H7 and H13 of the adjacent peptides can be spatially close in the dimer formed with the E11/H14:ZN:E11/H14 interface due to their antiparallel arrangement. We constructed the tetramer based on the H7/H13:ZN:H7/H13 interface, utilizing all four peptides from two dimers with the E11/H14:ZN:E11/H14 interface (Figure 8A). The resulting conformation of the D7H-Aβ_16_ tetramer with E11/H14:ZN:E11/H14 and H7/H13:ZN:H7/H13 interfaces was stable after 50 ns of MD modeling while maintaining the antiparallel orientation of the peptides. Its structure restricts further polymerization via the H7/H13:ZN:H7/H13 interaction interface, because the H7 and H13 residues not involved in the peptide interaction are far from each other. Extra polymerization was possible via a different interface, E3/H6:ZN:E3/H6. One octamer and one dodecamer with the E11/H14:ZN:E11/H14, E3/H6:ZN:E3/H6, and H7/H13:ZN:H7/H13 interfaces were constructed (Figure 9 and Figure 10).

As shown in Figure 10, the proposed equilibrium structure of the D7H-Aβ_16_ dodecamer has three polymerization seeds and can be easily extended for further polymerization. This is possible due to the presence of a third interface—H7/H13:ZN:H7/H13—in the D7H-Aβ_16_ molecule available for binding a Zn atom, while Aβ_16_ has only two interfaces. The alternation of different zinc coordination interfaces likely creates more stable and strongly bound structures of aggregates as there is a greater number of intermolecular bonds in such systems compared to simple stacking, such as in the aggregate shown in Figure 7.

### 2.5. Molecular Modeling of Zinc-Bound Heterotrimers of Aβ_16_/Zn/D7H-Aβ_16_/Zn/Aβ_16_ and Aβ_16_/Zn/isoD7-Aβ_16_/Zn/Aβ_16_

**Figure 11:**
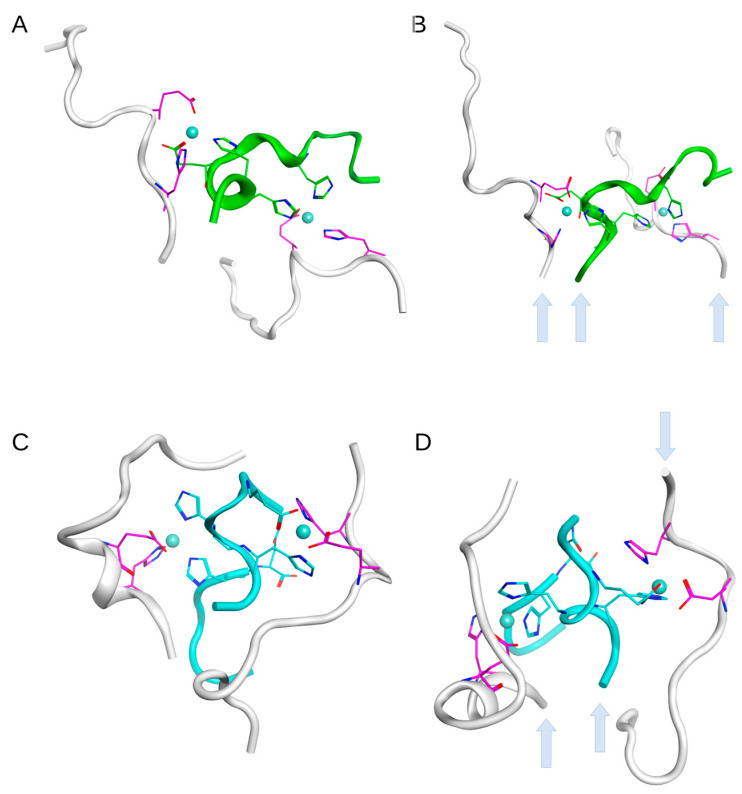
Equilibrium conformations of the trimers formed by D7H-Aβ_16_ (colored with green) with two standard Aβ_16_ peptides (**A**,**B**) and isoD7-Aβ_16_ (colored with cyan) with two standard Aβ_16_ peptides (**C**,**D**) obtained after 100 ns of MD. (**A**) interaction interface between D7H-Aβ_16_ and two standard Aβ_16_ peptides bound via Zn ions. Zn ions are coordinated with Glu11 and His14 residues of the standard Aβ_16_ peptides (colored with magenta) and with residues His7, His13 and Glu11, His14 of the D7H-Aβ_16_ peptide. (**B**)—different view on the complex presented in (**A**). C-termini are shown with arrows. (**C**) interaction interface between the isoD7-Aβ_16_ and two standard Aβ_16_ peptides bound via Zn ions. Zn ions are coordinated with Glu11 and His14 residues of the standard Aβ_16_ peptides (colored with magenta) and with residues His6, His13 and Glu11, His14 of the isoD7-Aβ_16_ peptide. (**D**) different view on the complex presented in (**C**). C-termini are shown with arrows.

The MD modeling presented in the previous section showed the variability and flexibility of the structures that grow due to zinc ions located in the coordination centers of D7H-Aβ_16_ peptides. The position of the residues 6 and 7 is critically important for the structure of the resulting complexes as it bears upon the activation of the zinc binding site that involves H13. In the Aβ_16_ peptide, the metal binding site H6/H13 is not present. The D7H mutation provides a new zinc ion coordination center, H7/H13. At the same time, the isomerization of the D7 residue rotates the N-terminus of the Aβ_16_ peptide and enables a coordination center, H6/H13, for the zinc ion [21]. We speculate that the D7H and isoD7 modifications can emerge in the population of Aβ_16_ and become the seeds of rapid aggregation through zinc binding interfaces created by an additional metal binding site in D7H-Aβ_16_ and isoD7-Aβ_16_, in contrast to Aβ_16_. To explore this possible mechanism in more detail, we created two systems of peptide trimers (Figure 11) bound through zinc ions.

A 50–100 ns MD modeling of single Aβ_16_, D7H-Aβ_16_, and isoD7-Aβ_16_ molecules was performed. The D7H-Aβ_16_ and isoD7-Aβ_16_ conformations where zinc ions can be simultaneously located in the two coordination centers—H7/H13 and E11/H14 for D7H-Aβ_16_; H6/H13 and E11/H14 for isoD7-Aβ_16_—were extracted from the 50 ns MD trajectories. For the Aβ_16_ molecules, conformations randomly extracted from the MD trajectory were arranged on both sides of a modified Aβ_16_ molecule (D7H-Aβ_16_ or isoD7-Aβ_1_) in such a way that their E11/H14 sites were located near the corresponding zinc binding sites of the central modified molecule (Figure 11). Then, zinc ions were placed in each coordination center, and the whole system was subjected to MD simulation. After a 100 ns MD modeling, all of the complexes remained stable. The results show that both Aβ_16_ modifications can lead to the formation of initial trimer structures that serve as seeds for further aggregation of Aβ. The modeling of the trimer based on the D7H-Aβ_16_ modification showed that all three peptides in the trimer were positioned in parallel to each other, while in the trimer with the isoD7-Aβ_16_ modification, the Aβ_16_ molecule that was bound through a zinc ion to the H6/H13 site appeared in the antiparallel orientation to the two other molecules.

## 3. Discussion

D7H-Aβ_16_ exhibits extremely high propensity to aggregate in the presence of zinc ions [26]. We had previously shown that fragment 1–7 of the D7H-Aβ (D7H-Aβ_7_) peptide, in the presence of zinc ions, forms very stable dimers with a unique interface [26]. In the current study, we investigated the molecular mechanism of the zinc-induced oligomerization of D7H-Aβ_16_. Using turbidimetry and DLS, it was shown that D7H-Aβ_16_ significantly exceeds Aβ_16_ in its ability to aggregate in the presence of zinc ions. The maximum particle size formed as a result of the interaction of D7H-Aβ_16_ with Zn^2+^ is almost three orders of magnitude larger than that for Aβ_16_ in the presence of Zn^2+^ (Figure 2). At the same time, D7H-Aβ_16_/Zn^2+^ aggregates remain soluble and stable in solution (Figure 1). Using a combination of ITC and MS methods, it was found that in the zinc-bound D7H-Aβ_16_ oligomers, each D7H-Aβ_16_ subunit has three zinc-dependent interfaces formed by amino acid pairs E3/H6, H7/H13, and E11/H14. It was shown that the primary binding site of zinc ion in the D7H-Aβ_16_ is the 11-EVHH-14 site, and the E11 and H14 residues of the interacting peptides form a symmetrical zinc-bound intermolecular interface of the oligomers. The E3/H6 and H7/H13 pairs form two more interfaces, utilized for the formation of D7H-Aβ_16_ oligomers and aggregates in the presence of zinc ions. Modeling showed that these three intermolecular interfaces can be efficiently combined in the process of polymer growth.

According to the known molecular mechanism of the zinc-dependent oligomerization of isoD7-Aβ_16_, the key role in this process is played by the 11-EVHH-14 region, which is a structural determinant of all Aβ isoforms (with the exception of ratAβ) [21]. However, for Aβ, this site does not have a high capacity to participate in the intermolecular interactions stabilized by the zinc ion common for the interacting subunits of the Aβ homodimer as the zinc ion is preferably coordinated by four chelating amino acid residues (H6, E11, H13, H14) inside the compactly folded metal-binding domain 1–16 [16]. In contrast, in the presence of chemical modifications (isoD7, pS8) or H6R mutation, the corresponding Aβ isoforms, compared to Aβ, are dynamically represented by a range of conformations, including compact and extended structures, allowing them to form the interface E11-H14/Zn/E11-H14 in the homodimers of these molecules. For isoD7-Aβ, in addition to the E11-H14 interface, the H6-H13 interface emerges [21]. It is this second interface—appearing due to the mobility of the main chain in the isoD7 region—that causes isoD7-Aβ zinc-dependent polymerization, which is not observed for Aβ, pS8-Aβ, and H6R-Aβ molecules [39].

The ability of isoD7-Aβ to carry two zinc ion binding sites leads to the assumption that the interaction of two Aβ molecules with a single isoD7-Aβ molecule can theoretically result in the formation of a trimer consisting of a central isoD7-Aβ molecule, in which there are two pairs of amino acids residues (E11-H14 and H6-H13), each of which is involved in the joint coordination of a single zinc ion with the E11-H14 pair from each intact Aβ molecule (Figure 11). It is noteworthy that, according to the MD data, D7H-Aβ is also able to form a trimer with two intact Aβ molecules in the presence of zinc ions in a manner similar to isoD7-Aβ (Figure 11). Such trimers can act as seeds for a chain reaction of the pathogenic aggregation of intact Aβ molecules in the presence of zinc ions.

To summarize, the D7H mutation leads to the emergence of a new mechanism for the zinc-dependent oligomerization of the Aβ metal-binding domain. There, the role of the E11/H14 interface as the primary zinc-mediated intermolecular interface is preserved, but the association constant for this site is increased by an order of magnitude, and instead of the H6/H13 interface found for isoD7H-Aβ_16_ zinc-bound oligomers, the two interfaces (E3/H6 and H7/H13) emerge. This mechanism explains why D7H-Aβ_16_ is much more susceptible to zinc-induced aggregation in comparison with such domains of other Aβ isoforms. Also, the findings confirm that 11-EVHH-14 represents a universal target for drugs aimed at suppressing the zinc-dependent oligomerization of various Aβ isoforms. In addition, using molecular modeling, the higher potential amyloidogenicity of D7H-Aβ compared to isoD7-Aβ as an exogenous zinc-dependent oligomerization seed was substantiated as a result of the higher ability of D7H-Aβ to form zinc-induced trimers with two Aβ molecules.

## 4. Materials and Methods

### 4.1. Amyloid Peptides

All synthetic peptides (purity > 98%, checked by RP-HPLC) were purchased from Biopeptide Co., LLC (San Diego, CA, USA). The C-termini of each peptide was protected with amide. The amino acid sequence of each peptide was confirmed on an ultra-high resolution Fourier transform ion cyclotron resonance mass-spectrometer Bruker 7T Apex Qe (Bruker Daltonics, Billerica, MA, USA) using a de novo sequencing approach based on collision-induced dissociation (CID) fragmentation. The lyophilized peptides were dissolved immediately before each experiment in the appropriate buffer at 25 °C. Buffer solutions were made using MQ water and filtered through 0.22-micron filter. The final peptide concentrations were determined through UV absorption spectroscopy using the extinction coefficient of 1450 M^−1^ cm^−1^ at 276 nm (from Tyr10 of Aβ).

### 4.2. Turbidity and DLS Measurements

The optical density (OD) of the peptide solutions was measured at 405 nm on a UV/VIS spectrophotometer Model V-560 (Jasco Corporation, Tokyo, Japan) in 50 mM Tris (USB Corporation, Cleveland, OH, USA), pH 7.3. A 1-mM solution of Aβ peptides was placed into a quartz cell with a path length of 10 mm and the OD values were collected at 25 °C. Measurements of the peptides in the absence of Zn^2+^ were carried out after 2 h, following the addition of buffer to the peptide. ZnCl_2_ was added to the peptide solutions at a concentration of 2.25 mM, and the optical density of the solutions was measured for 26 h. The OD_405_ values measured in the absence of Zn^2+^ were taken as the initial (zero time) values.

DLS measurements were carried out on a Zetasizer Ultra apparatus (Malvern Instruments Ltd., Malvern, UK) in 50 mM Tris, pH 7.3, at 25 °C, as described elsewhere [29,40]. The 100 μL aliquots of peptide solutions were placed into a BRAND UV microcuvetter (BRAND GMBH, Wertheim, Germany) and used for the measurements. Measurements of peptides in the presence of Zn^2+^ were carried out within 10 min after the addition of ZnCl_2_ to the peptide solutions. The instrument is equipped with a He-Ne laser source (λ = 632.8 nm) and operates in the back-scatting mode, measuring the particle size in the range between 0.3 nm and 10 μm. The characteristic size of Aβ aggregates was expressed in terms of the average “characteristic diameter” as the instrument software (ZS Xplorer 2.2.0.147) approximates the heterogeneous population of Aβ aggregates through a population of spherical particles with the identical distribution of the diffusion coefficient. The number of particles distributions were used by the instrument software to calculate the characteristic diameters of the particles.

### 4.3. Isothermal Titration Calorimetry

The thermodynamic parameters of zinc binding to D7H-Aβ_16_ and its mutants were measured using a MicroCal iTC200 System (GE Healthcare Life Sciences, Milwaukee, WI, USA), as described previously [31]. Experiments were carried out at 25 °C in 50 mM Tris buffer (USB Corporation, Cleveland, OH, USA), pH 7.3. Next, 2 µL aliquots of the ZnCl_2_ (Sigma-Aldrich, St. Louis, MO, USA) solution were injected into the 0.2 mL cell containing the peptide solution to obtain a complete binding isotherm. The peptide concentration in the cell was 0.3 mM and the ZnCl_2_ concentration in the syringe was 5 mM. The heat of dilution was measured by injecting the ligand into the buffer solution; the values obtained were subtracted from the heat of the reaction to obtain the effective heat of binding. The resulting titration curves were fitted using the MicroCal Origin 8 software. Affinity constants (K_a_), binding stoichiometry (N), and enthalpy (ΔH) were determined through a non-linear regression fitting procedure.

### 4.4. Mass-Spectrometry

All the experiments were performed on a Finnigan LTQ FT mass-spectrometer with an ESI ion source. Peptides were dissolved in a water-methanol solution with zinc acetate as the source for zinc ions. The content of the solution was varied in order to optimize the ionization conditions. In the experiments, the concentration of the peptides in the solution was varied between 200 nM and 10 μM, and the concentration of zinc ions between 200 nM and 2 mM. The MS spectra of the molecular ions of the amyloid peptides and their complexes with zinc ions in different charge states were obtained. Using two complementary techniques of fragmentation—electron capture dissociation (ECD) and collision induced dissociation (CID) methods—the fragmentation spectra of the zinc-bound complexes were measured and analyzed. The main zinc ion coordinators were deduced using a specially developed algorithm based on the analysis of the intersections of fragments carrying different amounts of bound zinc ions resulting from the fragmentation of various peptide-metal complexes.

### 4.5. Molecular Modelling

#### 4.5.1. Structure Preparation

The initial structures of Aβ_16_ and templates for D7H-Aβ_16_ were obtained from our previous work [39]. As a result of this work, we obtained more than 30 different variants of the Aβ_16_ dimer, in which the non-mutated peptides were zinc-linked at residues 11 and 14, and more than 10 structures of Aβ_16_ peptides in monomeric form. The D7H-Aβ_16_ structure was obtained through the manual point mutation of Asp7 to His of the Aβ_16_ peptides or dimers and subsequent relaxation through molecular dynamics for 50–100 ns with trajectory recording. The isoD7-Aβ_16_ structure was obtained through the manual point mutation of the Asp7 of the Aβ_16_ peptide and subsequent relaxation through molecular dynamics for 50 ns with trajectory recording.

#### 4.5.2. Molecular Modeling Protocol

MD simulations were carried out with the GROMACS 2022.3 software [41]. All models were first processed through an energy minimization procedure, sequentially, using the steepest descent and conjugated gradients algorithms until a local minimum was reached. Then, a two-stage equilibration of the system was carried out in NVT and NPT ensembles, each for 100 ps, respectively. In the simulation, the Ewald summation algorithm was used, the constraints on the motion of atoms were set using the LINCS algorithm. The cutoff radii of the Coulomb and Van der Waals potentials were 1.2 nm. The time step was 0.2 fs. All systems included periodic boundary conditions. Water and ions were modeled explicitly using the TIP3 model for water. For zinc ion and its coordinating residues, parametrization of the Procacchi et al. force field was applied [42].

## Figures and Tables

**Table 1:** Thermodynamic parameters of Zn^2+^ binding to Aβ_16_, D7H-Aβ_16_ and its mutants obtained by ITC at 25 °C ^a^.

	N ^b^	K_a_ ^c^ × 10^−4^, M^−1^	ΔH ^d^ kcal mol^−1^
Aβ_16_ ^e^	1.1	1.8	−4
D7H-Aβ_16_	1	2.3	−10.3
	0.35	48	−8.0
E11A-D7H-Aβ_16_	1.1	2.1	−8.7
H13A-D7H-Aβ_16_	1	1.7	−8.7
	0.3	20	−6.0
H14A-D7H-Aβ_16_	1	1.8	−9.0
D7H-Aβ_10_ ^f^	1	4.1	−10.4

^a^ All the measurements were performed three times in 50 mM Tris buffer, pH 7.3. ^b^ N—stoichiometry; standard deviation did not exceed ±15%. ^c^ K_a_—affinity constant; standard deviation did not exceed ±25%. ^d^ ΔH—enthalpy variation; standard deviation did not exceed ±20%. ^e^ Data from [36]. ^f^ Data from [26].

## Data Availability

The data are available upon request.
